# Work Participation and Executive Abilities in Patients with Relapsing-Remitting Multiple Sclerosis

**DOI:** 10.1371/journal.pone.0129228

**Published:** 2015-06-17

**Authors:** Karin van der Hiele, Dennis van Gorp, Rob Ruimschotel, Noëlle Kamminga, Leo Visser, Huub Middelkoop

**Affiliations:** 1 National MS Foundation, Maassluis, The Netherlands; 2 Department of Psychology, Section Health, Medical and Neuropsychology, Leiden University, Leiden, The Netherlands; 3 Medical Psychiatric Centre PsyToBe, Rotterdam, The Netherlands; 4 Department of Neurology, Leiden University Medical Centre, Leiden, The Netherlands; 5 Department of Neurology, St. Elisabeth Hospital, Tilburg, The Netherlands; 6 University of Humanistic Studies, Utrecht, The Netherlands; University of Oxford, UNITED KINGDOM

## Abstract

The majority of patients with Multiple Sclerosis (MS) are unable to retain employment within 10 years from disease onset. Executive abilities, such as planning, working memory, attention, problem solving, inhibition and mental flexibility may have a direct impact on the ability to maintain a job. This study investigated differences in subjective and objective executive abilities between relapsing-remitting MS patients with and without a paid job. We included 55 relapsing-remitting MS patients from a community-based sample (47 females; mean age: 47 years; 36% employed). Patients underwent neurological, cognitive and psychological assessments at their homes, including an extensive executive test battery. We found that unemployed patients had a longer disease duration (t(53)=2.76, p=0.008) and reported more organising and planning problems (χ^2^(1)=6.3, p=0.012), higher distractibility (Kendall’s tau-b= -0.24, p=0.03) and more cognitive fatigue (U=205.0, p=0.028, r=-0.30) than employed patients. Unemployed patients completed slightly less categories on the Wisconsin Card Sorting Test (U=243.5, p=0.042, r=-0.28). Possible influential factors such as age, educational level, physical functioning, depression and anxiety did not differ between groups. In conclusion, while relapsing-remitting MS patients without a paid job reported more executive problems and cognitive fatigue than patients with a paid job, little differences were found in objective executive abilities. Further research is needed to examine possible causal relations.

## Introduction

Unemployment rates of 55–58% have been observed among patients with Multiple Sclerosis (MS) posing a high socio-economic burden [1, 2]. MS is mostly diagnosed in relatively young adults-between 20 and 40 years old- who are in the beginning or the midst of their working careers. In younger MS patients, work participation is associated with a better self-rated physical and mental health [3].

Cognitive impairment is a common symptom in MS affecting as many as 43% to 65% of MS patients during their lifetime [4–6]. Studies have shown that various aspects of cognitive functioning can be impaired in patients with MS, including attention, information processing efficiency, general executive functioning, processing speed and long-term memory [7]. These cognitive impairments can seriously disrupt a person’s professional and social life and negatively influence quality of life [5, 7–9]. Executive abilities are particularly important for daily functioning, including work ability, and the level of independent living arrangements possible for people diagnosed with MS [10, 11]. Drew and colleagues [11] revealed below average performance on some aspect of executive functioning (e.g. shifting, fluency, planning, reasoning and abstract thinking) in the majority of their community-based sample of MS patients. They found widespread executive difficulties in 17% of the MS patients.

Measures of conceptual reasoning, set shifting, problem-solving, attention, multi-tasking ability and processing speed were found predictive of employment status in MS [12–15]. Among other functional measures (i.e. disease characteristics, physical disability, fatigue, cognitive function, personality traits, mood disorder, and behavioral dysfunction) tests of executive functioning were particularly sensitive in discriminating employed versus disabled MS patients [13]. All of the above mentioned studies include patients with relapsing-remitting as well as the progressive subtypes of MS [12–15]. However, disease course has an impact on both employment status [13, 14] and executive functioning [11].

The current study aims to examine the relation between subjective and objective executive functioning and employment status in MS patients with a relapsing-remitting disease course. By focussing on this subgroup of patients, the effects of disease course are minimized and factors which are specifically important for work participation in relapsing-remitting MS patients will be highlighted.

## Materials and Methods

### Patients

The ‘MS Cognition Study’ is a large-scale inventory of cognitive and psychological problems in a community-based cohort of Dutch MS patients [16]. The first phase concerned an inventory of cognitive and psychological complaints by means of questionnaires amongst 718 patients with MS. In the second study phase, an equal number of MS patients with and without cognitive complaints were randomly selected to undergo cognitive and neurological assessments (N = 128 in total). Patients were excluded in case of a severe psychiatric disorder, history of alcohol/drugs abuse, history of traumatic brain injury with residual symptoms, exacerbation during the last 4 weeks, and in case of a cognitive examination in the six months prior to the study, leaving 117 patients. The current study included only relapsing-remitting MS (RRMS) patients, aged 18–64 years old, and with available employment data, leaving 55 patients.

### Ethics

The study was approved by the Medical Ethical Committee of the St. Elisabeth Hospital in Tilburg, The Netherlands. All subjects provided written informed consent.

### Demographic and disease characteristics

We gathered data on demographic and disease characteristics. Employment status was dichotomized as ‘employed’ or ‘unemployed’. The employed group consisted of patients who had a paid job, either full-time (35 or more hours per week), part-time (12–34 hours per week) or less than 12 hours a week (criteria Dutch Central Bureau of Statistics). The unemployed group consisted of patients without a paid job, including homemakers, volunteers, patients receiving disability or unemployment benefits, patients on prolonged medical leave, or early retirement.

### Cognitive assessment

#### Subjective executive functioning

The cognitive assessment started with a structured questionnaire to ascertain the presence, severity and impact of cognitive deficits, including organising and planning deficits. The Behavioural Assessment of the Dysexecutive Syndrome-Dysexecutive Questionnaire (BADS DEX) [17] was used to obtain self-ratings of executive functioning in the cognitive domain (e.g. ‘I have difficulty thinking ahead or planning for the future’).

#### Objective executive functioning

Premorbid intelligence was determined using the National Adult Reading Test (NART) [18]. A battery with tests of executive functioning and processing speed was administered. The Trail Making Test (TMT) [19], Stroop Colour Word Test (SCWT) [20], Paced Auditory Serial Addition Test [21] and the Wisconsin Card Sorting Test (WCST) [22] provided measures of attention, processing speed, cognitive flexibility and abstract thinking. The copy subtest of the Rey Complex Figure Test (RCFT copy) [23] provided a measure of attention, organisational and planning skills. The BADS [17] provided ecologically valid measures of planning, organisation, problem solving and attention.

### Psychological assessment

Self-report measures of anxiety and depression were obtained using the Hospital Anxiety and Depression Scale (HADS) [24]. The Fatigue Impact Scale was used to measure impact of MS-related fatigue on daily life [25] resulting in scores for physical, cognitive and social fatigue.

### Statistical analysis

SPSS for Windows (release 21.0) was used for data analysis. We examined differences in demographic and disease characteristics, cognitive and psychological functioning between employed and unemployed patients using parametric and non-parametric tests where appropriate. T-scores ≤ 30 and percentile scores ≤5 were considered below average. The level of statistical significance was set at p ≤ 0.05.

## Results

### Demographic and disease characteristics of the study sample

The study sample consisted of 55 RRMS patients of which 36% (N = 20) had a paid job (15% full-timers, 55% part-timers, and 25% working less than 12 hours per week). Patients without paid employment had a longer disease duration (U = 198.500, p = 0.008, r = -0.36) than patients with paid employment (Table 1).

**Table 1:** Demographic and disease characteristics of the study sample.

		Patients with paid employment (N = 20)	Patients without paid employment (N = 35)
age (years)	Mean (SD)	44.8 (5.6)	48.5 (8.3)
age at diagnosis (years)	Mean (SD)	36.3 (6.3)	35.3 (8.8)
time since diagnosis (years)	Mean (SD)	9.2 (5.0)	14.0 (6.7)*
white versus blue collar work	%	67%	n.a.
educational level^a^	Mean (SD)	5.4 (0.7)	5.5 (0.7)
working hours per week	Mean (SD)	19.1 (11.1)	n.a.
unpaid work outside home	N (%)	7 (35%)	20 (57%)
physical functioning(EDSS)	Mean (SD)	3.4 (1.4)	3.7 (1.2)

EDSS: Expanded Disability Status Scale;

^a^Educational level ranged from 1 (elementary school not completed) to 7 (university);

*p< = 0.05.

### Subjective executive functioning

Patients with and without paid employment differed in self-reported organising and planning problems (χ²(1) = 6.3, p = 0.012) (Table 2). While only 25% of the employed patients reported organising and planning problems, 60% of the unemployed patients reported this type of problem. When reported, the organising and planning problems were of moderate severity and impact. No group differences were found in self-reported executive problems on the BADS DEX cognitive scale. When looking into the five cognitive items of the BADS DEX in more detail, patients without paid employment more often reported difficulties in ‘sustaining attention and being easily distracted’ than patients without paid employment (Kendall’s tau-b = -0.24, p = 0.03).

**Table 2:** Subjective and objective executive functioning.

	Patients with paid employment (N = 20)	Patients without paid employment (N = 35)
**Subjective executive functioning**		
Organising and planning problems	25%	60%*
BADS DEX cognitive scale (total score; max 20)	5.8 (3.0)	7.2 (3.7)
**Objective executive functioning**		
NART (IQ)	106.4 (7.8)	106.5 (8.4)
TMT part a (s)	34.2 (10.5)	35.3 (15.0)
TMT part b (s)	77.0 (39.1)	83.9 (45.2)
SCWT word reading (s)	48.6 (7.0)	51.7 (10.5)
SCWT colour naming (s)	60.7 (9.5)	63.6 (12.2)
SCWT colour-word naming (s)	89.3 (15.7)	100.2 (29.8)
SCWT (interference t-score)	56.5 (9.3)	52.9 (8.3)
RCFT copy (percentile)	3.5 (1.7)	3.0 (1.6)
PASAT 3’(correct; max 60)	46.0 (10.2)	44.5 (12.2)
WCST (categories; max 6)	5.7 (0.8)	4.8 (1.6)*
WCST (perseverative responses)	15.7 (12.0)	17.5 (11.0)
BADS total score (profile score; max 24)	18.0 (3.4)	17.8 (3.2)

Means (SD) or percentages are listed in this table. BADS: Behavioural Assessment of the Dysexecutive Syndrome, DEX: Dysexecutive Questionnaire, NART: National Adult Reading Test, TMT: Trail Making Test, SCWT: Stroop Colour Word Test, RCFT: Rey Complex Figure Test, PASAT: Paced Auditory Serial Addition Test, WCST: Wisconsin Card Sorting Test.

*p< = 0.05.

### Objective executive functioning

Patients with paid employment completed more categories on the WCST than patients without paid employment (U = 243.5, p = 0.042, r = -0.28). There were no group differences in premorbid intelligence or in other aspects of executive functioning. We observed below average executive performance in 4% and 9% of the RRMS patients (based on TMT A and B), in 16%, 6% and 2% of the patients (based on SCWT word reading, colour naming and colour word naming), 51% (RCFT copy), 20% (PASAT score <32), 4% (WCST categories), 4% (WCST perseverative responses) and 6% (BADS profile impaired).

### Psychological characteristics

Patients without a paid job experienced higher levels of cognitive fatigue than patients with a paid job (U = 205.0, p = 0.028, r = -0.30). Self-reported depression, anxiety, physical and social fatigue did not differ between groups (Table 3).

**Table 3:** Psychological characteristics.

	Patients with paid employment (N = 20)	Patients without paid employment (N = 35)
HADS depression	5.3 (4.1)	5.4 (3.5)
HADS anxiety	7.1 (4.2)	6.6 (3.8)
FIS physical fatigue	15.1 (8.9)	20.3 (10.5)
FIS cognitive fatigue	13.5 (11.5)	20.7 (11.0)*
FIS social fatigue	27.5 (17.6)	35.1 (17.4)

Means (SD) are listed. HADS: Hospital Anxiety and Depression Scale, FIS: Fatigue Impact Scale;

*p< = 0.05.

The raw data concerning demographics, disease characteristics, subjective and objective executive functioning, and psychological characteristics can be found in the supporting information (S1 Data).

## Discussion

This study examined whether subjective and objective executive abilities differ between RRMS patients with and without a paid job. Executive functions refer to abilities such as planning, working memory, attention, problem solving, inhibition and mental flexibility. These abilities are often impaired in patients with MS and may have a direct impact on the ability to maintain a job. Contrary to our expectations we observed only minor differences in objective executive abilities between RRMS patients with and without a paid job, i.e. a slightly decreased set shifting ability as measured with the number of completed categories on the WCST. It should be noted that only 4% of the RRMS patients performed below average on this measure. The WCST has been associated with vocational status in previous research [12, 13] although specifically pertaining to the number of perseverations. The PASAT, a test of mental flexibility, information processing speed, working memory and inhibition, did not differ between groups even though this was observed in the study of Honarmand et al. (2011) [26]. No group differences were found using the ecologically valid BADS battery.

It should be noted that below average performance was observed in 2–55% of the patients on some aspect of executive functioning, with the highest percentages related to decreased processing speed as measured with the PASAT (in 20% of the patients) and impaired performance on the RCFT copy (in 51%). The found impairments in copying the Rey-Osterrieth-Figure might be related to difficulties with various cognitive processes, including attention and concentration, visuospatial perception and processing visual information, visual-motor function and planning and organisational skills [27]. In summary, few differences in executive abilities were detected between MS patients with and without a paid job. As many patients without a paid job (57%) kept working outside their homes, e.g. by doing volunteer work, their overall executive abilities may remain intact.

### Subjective executive problems and fatigue

Despite little differences in objective executive performance, we found a much higher prevalence of subjective organising and planning problems in unemployed versus employed MS patients (i.e. in 60% versus 25%). Furthermore, unemployed patients reported being easily distracted and having difficulties sustaining attention on a more frequent basis. Depression and anxiety have been previously linked to a negative report bias [16, 28]. As we observed no relation between mood and employment status, psychological factors may not be related to the increased subjective problems in unemployed versus employed MS patients. Unemployed MS patients reported a larger impact of fatigue on cognitive activities. Fatigue has been previously associated with unemployment in MS [1, 29, 30]. Self-reports of cognitive fatigue may be related with self-reported cognitive impairment [31]. Interestingly, a recent study in MS patients found fatigue to be related to subjective memory problems, but not memory impairment [32].

As in our previous work [16] we observed that self-reports of cognitive performance and the influence of fatigue on cognitive performance may not always be related to objective cognitive difficulties. As these self observed difficulties may have a large impact on important life decisions an effort should be made to properly identify both subjective and objective cognitive problems.

### Strengths and limitations

Strengths of the current study include its focus on patients with RRMS. Previous studies also included progressive subtypes and found that disease course has a large effect on both employment status and executive functioning. By focussing on this particular group, factors that may be more specific to unemployment in RRMS patients are highlighted, such as (perceived) cognitive changes, mood and fatigue. By using an ecologically valid executive test battery we included tests sensitive for everyday organising and planning problems that influence work ability. Unfortunately our executive test battery did not include the Symbol Digit Modalities Test. Performance on this test has been associated with work participation in previous MS studies [13, 26, 33].

A main limitation of this study is its cross-sectional design and the lack of a control group, which makes it impossible to make any causal inferences. No information was available about the past work situation and whether MS-related disability was the main reason for unemployment. The found employment rate of 36% is nevertheless lower than in the total Dutch population in 2010, with rates varying from 66% (15–39 years old) to 80% (40–54 years old) and then dropping to 49% in people aged 55 years and older [34].

### Clinical implications

In this study, we observed little to no relations between objective executive abilities and employment status in RRMS patients. MS patients without a paid job did report more problems with organising and planning skills and mentioned increased distractibility and cognitive fatigue. They had been diagnosed for a longer period of time. One interpretation of our findings is that self-perceived cognitive problems and related fatigue may be part of the reason that MS patients decide not to pursue a working career. Identification of both subjective and objective cognitive difficulties and fatigue may be of particular importance in rehabilitation or prevention of work problems, e.g. by offering adaptions at work (e.g. the use of organising and planning tools or changes in the working schedule) or by psychological interventions aimed at coping with the experienced cognitive difficulties.

Longitudinal research is needed to further examine the relation between disease characteristics, subjective and objective cognitive abilities and work activities in patients with MS.

## Supporting Information

S1 Data Raw data. This file contains the data on demographics, disease information, executive and psychological functioning for this study on Work Participation and Executive Abilities in Patients with Relapsing Remitting Multiple Sclerosis.
(XLSX)
